# EDITOR’S MESSAGE / MESSAGE DE LA RÉDACTION

**DOI:** 10.29173/jchla29976

**Published:** 2026-08-03

**Authors:** Ashley Farrell

**Affiliations:** JCHLA/JABSC Editor

[*Le français suit*]

Happy summer everyone and welcome to the August 2026 issue of JCHLA/JABSC. I hope everyone is able to stay cool and enjoy the warm weather.

This issue opens with a research article from Ostapyk, Askin, Epp, & Tichon on assessing client engagement with literature search results titled, *When patrons “click” with the library’s literature searches: using data from a novel link generation process to assess patron engagement with literature search results*.

In this issue, we include a book review from Mason on the text, *Knowing our value and our values: toward an ethical practice of library assessment*. We have three product reviews; Hilton considers the graphic medicine resource *GraphicMedicine.org*, Kitchin examines the screening and review management software *Rayyan.ai*, and Martyniuk and McNeil analyze the AI-powered clinical decision support tool *OpenEvidence*.

Our issue concludes with the abstracts from the CHLA/ABSC 2026 conference that took place in beautiful Québec City this past June. I hope you will take the time to peruse the abstracts for the many great papers, posters, lightning talks, interactive workshops, and panels that made up the program this year. If you did present at CHLA/ABSC this year—please consider turning your conference presentation into a manuscript! JCHLA/JABSC would be happy to support you in publishing your research.

This August issue marks the end of my three-year term on the JCHLA/JASBC Editorial Board. I have found this to be a rewarding and enrichening experience. I am grateful to the rest of the Editorial Board for all of their support and hard work. In September, Melissa Walter (Canada’s Drug Agency) will move into the Editor role and Katie Merriman (McMaster University) will become the Senior Editor. Tyler Ostapyk (University of Manitoba) will stay on as Production Editor and Kate Merucci (Health Canada) will continue as Copyeditor. We are happy to welcome Talin Boghosian (Unity Health Toronto) to the team as the new Junior Editor.

Thank you to all of our authors and peer reviewers for their contributions and thank you to our readers for your continued support.

**Ashley Farrell**

*JCHLA/JABSC Editor*

*Email:* editor@chla-absc.ca

Bon été à tous et bienvenue dans le numéro d’août 2026 de JABSC/JCHLA. J’espère que vous parvenez tous à rester au frais et à profiter de la chaleur.

Ce numéro s’ouvre sur un article de recherche d’Ostapyk, Askin, Epp et Tichon portant sur l’évaluation de l’engagement des usagers vis-à-vis des résultats de recherche documentaire, intitulé « Quand les usagers “cliquent” avec les recherches documentaires de la bibliothèque : utilisation des données issues d’un nouveau processus de génération de liens pour évaluer l’engagement des usagers vis-à-vis des résultats de recherche documentaire ».

Dans ce numéro, nous incluons une critique de livre de Mason sur l’ouvrage « Connaître notre valeur et nos valeurs : vers une pratique éthique de l’évaluation des bibliothèques ». Nous proposons trois critiques de produits : Hilton se penche sur la ressource de médecine graphique GraphicMedicine.org, Kitchin examine le logiciel de filtrage et de gestion des revues Rayyan.ai, et Martyniuk et McNeil analysent l’outil d’aide à la décision clinique alimenté par l’IA, OpenEvidence.

Notre numéro se termine par les résumés de la conférence CHLA/ABSC 2026 qui s’est tenue dans la belle ville de Québec en juin dernier. J’espère que vous prendrez le temps de parcourir les résumés des nombreux excellents articles, affiches, présentations éclair, ateliers interactifs et tables rondes qui composaient le programme cette année. Si vous avez fait une présentation à la CHLA/ABSC cette année, pensez à transformer votre présentation de la conférence en article! La JCHLA/JABSC se fera un plaisir de vous aider à publier vos travaux de recherche.

Ce numéro d’août marque la fin de mon mandat de trois ans au sein du comité de rédaction de la JCHLA/JASBC. Cette expérience s’est avérée enrichissante et valorisante. Je tiens à remercier les autres membres du comité de rédaction pour leur soutien et leur travail acharné. En septembre, Melissa Walter (Agence des médicaments du Canada) assumera le rôle de rédactrice en chef et Katie Merriman (Université McMaster) deviendra rédactrice en chef adjointe. Tyler Ostapyk (Université du Manitoba) conservera son poste de rédacteur de production et Kate Merucci (Santé Canada) continuera d’occuper celui de réviseure. Nous sommes heureuses d’accueillir Talin Boghosian (Unity Health Toronto) au sein de l’équipe en tant que nouvelle rédactrice adjointe.

Merci à tous nos auteurs et évaluateurs par les pairs pour leurs contributions, et merci à nos lecteurs pour leur soutien continu.

**Ashley Farrell**

*Directrice de la rédaction JABSC/JCHLA*

*Courriel:* editor@chla-absc.ca

